# Enlisting Genomics to Understand Flu Evolution

**DOI:** 10.1371/journal.pbio.0030302

**Published:** 2005-07-26

Last October, as Americans started lining up for flu shots, news broke that 48 million vaccine doses had been contaminated. With 100 million people considered at high risk and fears of a potentially deadly avian flu epidemic on the horizon, the shortage caused long lines, allegations of price gouging, and a new bill to bolster the nation's anemic vaccine manufacturing base.

Influenza A viruses are RNA viruses that infect humans, pigs, horses, and birds, both wild and domestic. Flu infection relies on a viral glycoprotein, hemagglutinin (HA), that binds to receptors on a host cell and allows the virus to be internalized. If antibodies produced by host immunity recognize viral antigens (on the surface of the HA protein), HA binding is inhibited and infection prevented. A virus's best chance of gaining the upper hand in this evolutionary game of cat and mouse is to change its HA in a way that eludes antibody recognition. Typically the mutations are minor and the virus's antigens conserved enough for the host body's immune system to recognize them. On occasion, influenza can acquire an antigenically novel HA subtype, becoming a virulent pandemic strain that completely escapes immune surveillance and kills millions. Minimizing the effect of yearly influenza outbreaks—by developing effective matched vaccines—depends on predicting which flu strains are likely to evolve.

Toward this end, Eddie Holmes and colleagues took the global approach afforded by genomics to explore the forces underlying viral adaptations. They found multiple flu strains circulating in the population at the same time, and a more complex evolutionary pattern than previously thought. They also showed that co-circulating viruses can exchange genes in a way that creates antigenically novel, epidemiologically significant strains—a process that humans may facilitate by simultaneously hosting more than one strain. [Fig pbio-0030302-g001]

**Figure pbio-0030302-g001:**
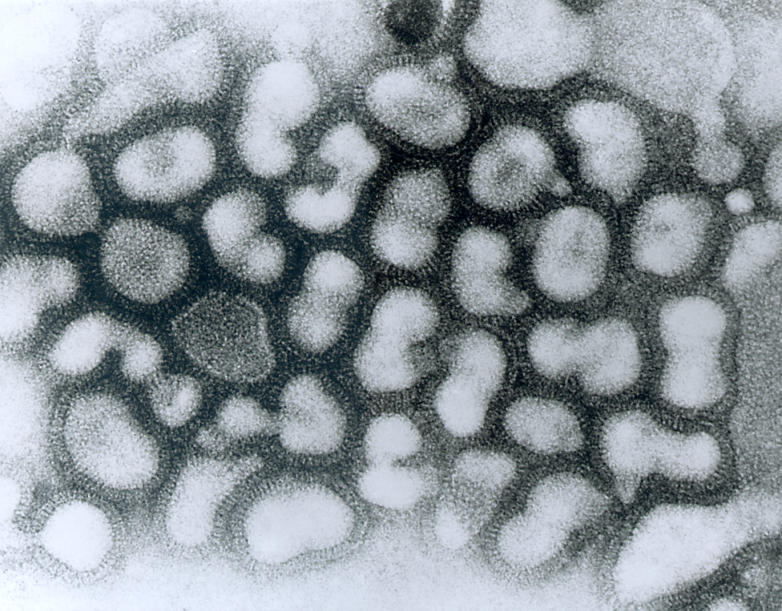
A transmission electron micrograph of the influenza A virus. New evidence suggests that flu viruses can rapidly reshuffle genetic material and mutate into new strains capable of widespread infection. (CDC/Dr. Erskine Palmer)

They analyzed the genomes of 156 influenza A viruses (serotype H3N2) collected by New York State public health officials between 1999 and 2004 in search of global patterns of viral evolution. Using the flu virus genome sequences produced at the Institute for Genomic Research (TIGR), funded by a National Institute for Allergy and Infectious Diseases (NIAID) initiative, the authors grouped the viral sequences according to sequence similarity. They also included partial flu sequences obtained from other studies in their analysis. These data are the initial output of the first large-scale effort to completely sequence influenza genomes. While most of the virus genomes sampled after 2002 fell into one group—which the authors called clade A—there were also other clades circulating at different times (called clades B and C).

Gene trees, or phylogenies, constructed for each of the virus's eight genes all diverged according to their respective clades, except one—the HA gene. The HA gene cluster grouped all the clade A viruses that emerged after 2002 as well as both the clade B and C viruses from the same time period and viruses from multiple locations (in Asia, Australia, Europe, and North America).

Altogether, these results indicate that different viral strains had circulated in the same populations until 2002 and then the clade A and C viruses acquired a common HA gene from clade B through reassortment. While reassortment between co-circulating human influenza strains has been previously described, this study is the first to examine in detail a reassortment event leading to an epidemiologically significant outcome, the emergence of the “Fujian” strain in the 2003–2004 season. Though it's not yet clear how variant clades manage to persist alongside dominant strains, the fact that they do suggests the influenza virus has multiple adaptive tools at its disposal. Luckily, the tools of genomics should help predict what evolutionary paths the virus might take and help in the process of selecting the most promising vaccines to contain it.

